# Supplementary material for: Reversed Polarity bi-tDCS over M1 during a Five Days Motor Task Training Did Not Influence Motor Learning. A Triple-Blind Clinical Trial
Source: Brain Sci. 2021 May 25;11(6):691. doi: 10.3390/brainsci11060691 (PMC8225177; doi:10.3390/brainsci11060691)
Supplement: Supplementary file 1 [file brainsci-11-00691-s001.zip › brainsci-1193026-supplementary.pdf]

Supplementary material.

Table 1. Descriptive analysis of sociodemographic data (n = 23). To evaluate the homogeneity of the different variables between groups. Independent Student's t-test was used for quantitative variables (mean ± standard deviation) and Pearson's chi² for categorical variables (number of subjects and percentage).

Variables	Bi-RP-tDCS group (n = 13)	Sham group (n = 10)	p-value
**Quantitative**			
Age	26.8 ± 5.3	23 ± 1.3	0.025
Sleep Quality	4 ± 2.1	6.7 ± 2.9	0.026
Physical activity	8485.2 ± 5620.3	6220.4± 3939.2	0.337
Edinburgh Inventory	19.8 ± 3.9	18.6 ± 3.3	0.760
**Qualitative**			
Gender			
- Women	5	8	0.046
- Man	8	2	
Educational level			
- High school	3	0	0.103
- University	10	10	

Table 2. Motor learning during bi-RP-tDCS. (mean ± S.D) n =23 for fine motor dexterity and n = 20 for gross motor dexterity.

Motor Dexterity		Group	Basal condition	Post first training	Post training	Post 5 days without training	Linear Mixed Model					
							Time		Group		Time*Group	
							F	p-value	F	p-value	F	p-value
Fine n = 23	Unimanual	Bi-RP-tDCS	16.9 ± 1.3	18.7 ± 1.6	20.6 ± 1.6	20.4 ± 1.4	30.9	<0.001	5.8	0.018	.4	.765
		Placebo	16.5 ± 1.7	18.7 ± 1.7	19.7 ± 1.7	19.3 ± 1.2						
	Bimanual	Bi-RP-tDCS	13 ± 1	13.6 ± 0.9	14.2 ± 0.9	14.5 ± 1.2	9.9	<0.001	0.9	0.353	.4	.790
		Placebo	12.7 ± 1.4	13.7 ± 1.6	14.3 ± 1.8	14.4 ± 1.3						
Gross n = 20	Unimanual	Bi-RP-tDCS	62.4 ± 4.6	56.9 ± 3.3	55.6 ± 3.9	55.8 ± 4.1	11.6	<0.001	4.8	0.032	.2	.879
		Placebo	63.2 ± 4.5	60.8 ± 4	58.4 ± 3.6	57.3 ± 3.9						
	Bimanual	Bi-RP-tDCS	46.9 ± 2.4	41.4 ± 2	40 ± 1.4	38.8 ± 2.3	34.5	<0.001	11.3	0.001	.2	.884
		Placebo	49.5 ± 4.8	43.6 ± 3.9	42 ± 3.5	41 ± 2.8						

Table 3. Motor training and bi-RP-tDCS effects on grip strength (mean ± SD), n = 23.

Variable	Group	Basal condition	Post first training	Post training	Post 5 days without training	Linear Mixed Model					
						Time		Group		Time*Group	
						F	p-value	F	p-value	F	p-value

Grip strength	**Bi-RP-tDCS**<br>**Placebo**	31.6 ± 8.6<br>27.5 ± 9.6	33.1 ± 8.6<br>29.2 ± 10.5	34.2 ± 9.7<br>29.9 ± 11.8	35.2 ± 9.7<br>28.9 ± 9.2	.2	.921	5.7	0.019	.1	.947

Table 4. Training and bi-RP-tDCS effects on sensory variables (mean ± SD), n = 23.

**Sensory thresholds**	**Group**	**Basal condition**	**Post first training**	**Post training**	**Post 5 days without training**	**Linear Mixed Model**					
						**Time**		Group		Time*Group	
						F	p-value	F	p-value	F	p-value
Two-point discrimination	**Bi-RP-tDCS**<br>**Placebo**	12.7 ± 6.4<br>11.8 ± 5.5	11.7 ± 6.3<br>11.3 ± 6	11.5 ± 4.9<br>8.8 ± 4.5	10.7 ± 6.5<br>8.8 ± 5.7	1.6	.184	2.3	0.132	.2	.882
Mechanical detection	**Bi-RP-tDCS**<br>**Placebo**	.027 ± .012<br>.024 ± .01	.026 ± .011<br>.027 ± .017	.029 ± .014<br>.028 ± .016	.033 ± .014<br>.023 ± .012	.1	.959	1.3	0.265	.5	.668
Thenar pressure pain	**Bi-RP-tDCS**<br>**Placebo**	5.9 ± 1.9<br>5.6 ± 1.7	5.9 ± 2<br>5.4 ± 1.5	5.9 ± 1.8<br>5.3 ± 1.7	6.4 ± 1.7<br>5.6 ± 1.6	.3	.840	2.2	0.137	.1	.951
Bone pressure pain	**Bi-RP-tDCS**<br>**Placebo**	7.2 ± 3<br>6.9 ± 2.3	6.8 ± 2.9<br>7.2 ± 2.9	7.7 ± 2.6<br>7.1 ± 2.8	7.8 ± 2.8<br>7.9 ± 3.4	.2	.884	0.001	0.982	.3	.851

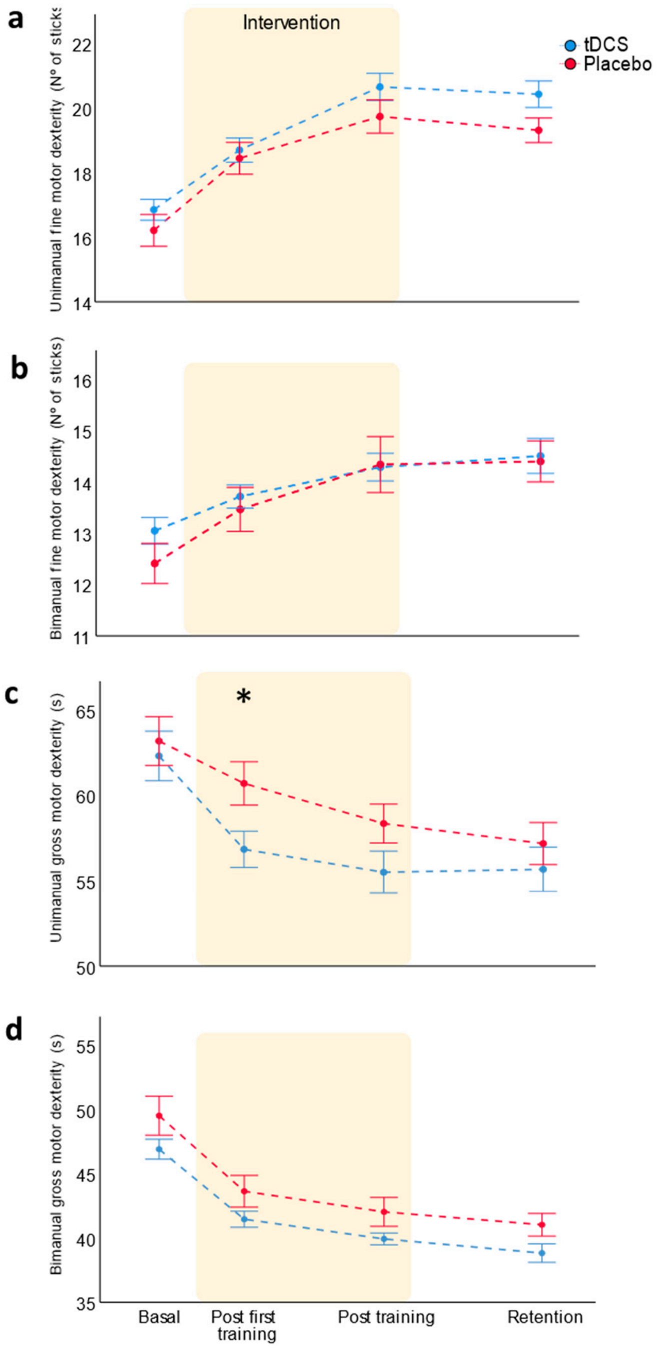

Figure S1. Bi-RP-tDCS effects on motor learning training. This figure shows the temporal evolution of the different motor dexterity variables assessed along days: (**a**) unimanual and (**b**) bimanual fine motor dexterity (n = 23); (**c**) unimanual and (**d**) bimanual gross motor dexterity (n =20). The data representation shows mean ± SEM.

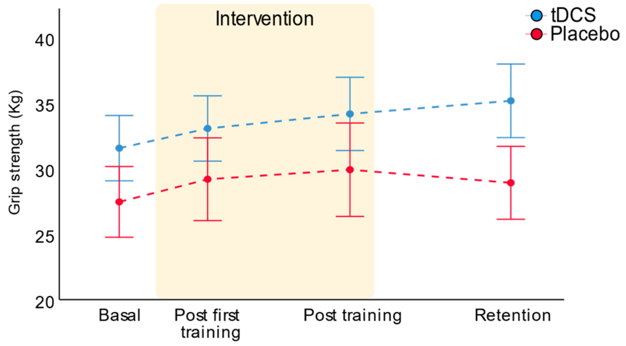

Figure S2. Bi-RP-tDCS effects on grip strength (n = 23).

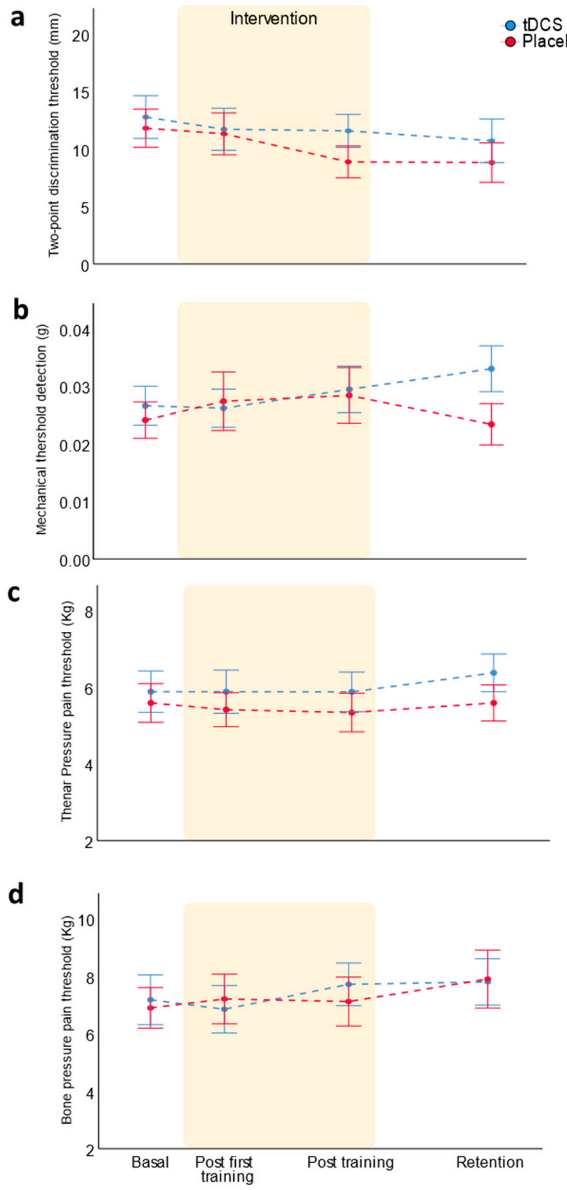

Figure S3. Bi-RP-tDCS and training effects on hand sensitivity along days (n = 23). (**a**) two-point discrimination; (**b**) mechanical detection threshold; (c) pressure pain threshold in the thenar eminence; (**d**) pain pressure pain threshold in the diaphysis of the second metacarpal bone. The data representation shows mean ± SEM.
